# Continuous Uptake of MiR-181a-2 Mimetic Induces Constitutive Overexpression of Cellular MiR-181a-2 in MCF-7 Breast Cancer Cells: Links with Progression of Drug Resistance

**DOI:** 10.34172/apb.025.43440

**Published:** 2025-08-26

**Authors:** Olga Evgen’evna Andreeva, Danila Vladimirovich Sorokin, Svetlana Vladimirovna Vinokurova, Pavel Borisovich Kopnin, Nadezhda Viacheslavovna Elkina, Danila Sergeevich Elkin, Maria Dmitrievna Fedorova, Alexander Mikhailovich Scherbakov, Mikhail Aleksandrovich Krasil’nikov

**Affiliations:** ^1^N.N. Blokhin National Medical Research Center of Oncology, the Ministry of Health of Russia, Moscow, Russia; ^2^Gause Institute of New Antibiotics, Moscow, Russia

**Keywords:** Breast cancer, MCF-7, MicroRNA, MiR-181a-2, Snail, Tamoxifen resistance

## Abstract

**Purpose:**

The aim of this study was to elucidate the mechanisms underlying the formation and maintenance of drug resistance in cancer cells. Previously, we demonstrated that prolonged treatment of estrogen-dependent MCF-7 breast cancer cells with exosomes derived from estrogen-resistant MCF-7/T cells leads to a partial loss of estrogen sensitivity in MCF-7 cells. Moreover, repeated transfection with one of the exosomal microRNAs—microRNA-181a-2—induced an irreversible decrease in hormonal sensitivity in the recipient cells. In the present work, to further investigate the possible mechanism of miR-181a-2-induced acquired resistance, we analyzed the effect of multiple miR-181a-2 transfections on the expression of cellular miR-181a-2 and related signaling proteins.

**Methods:**

miR-181a-2 was ectopically expressed by mimetic transfection or suppressed by antisense oligonucleotides. miR-181a-2 precursor/MIR181A2HG expression (qRT-PCR) and MIR181A2 locus copy number (qPCR) were assessed. wtSnail was expressed via transient transfection. Tamoxifen sensitivity was measured by MTT assay. Protein expression was studied by immunoblotting, estrogen receptor α/Snail transcriptional activity was evaluated by reporter analysis.

**Results:**

We found that multiple transfections with miR-181a-2 resulted in a marked increase in cellular miR-181a-2 precursor levels, whereas single transfection had no such effect. Similarly, stable transfection with miR-181a-2 led to increased levels of cellular miR-181a-2 and its host gene, MIR181A2HG, which was associated with partial resistance to tamoxifen. Analysis of the genomic DNA encoding miR-181a-2 revealed no changes in copy number in transfected cells. Furthermore, we identified the transcription factor Snail as a key mediator of miR-181a-2–induced resistance and demonstrated its role in the formation of an autoregulatory loop of miR181a-2 and the maintenance of cell resistance.

**Conclusion:**

Overall, these results reveal a novel mechanism of resistance-associated signaling pathway rearrangement based on the formation of a miR-181a-2 autoregulatory loop.

## Introduction

 Despite the proven effectiveness of hormone therapy, its clinical application is limited by the development of tumor resistance to hormones, which can be either primary or acquired, i.e., emerging during hormonal treatment.^1^ The mechanisms underlying hormonal resistance are well studied: resistance can arise due to an irreversible blockade of hormonal signaling (typically via suppression of the activity or expression of specific intracellular hormone receptors) or via activation of growth-regulating signaling pathways that bypass hormone-dependent signaling.^2-5^

 In addition to reduced receptor levels, major factors contributing to hormonal resistance include: (i) an imbalance between receptor coactivators and corepressors; (ii) ligand-independent receptor activation; and (iii) stimulation of hormone-independent growth pathways (primarily mediated by tyrosine kinase receptors) that sustain tumor growth in the absence of hormones.

 Primary resistance is typically associated with mutations in specific genes that disrupt hormonal signaling (e.g., repression of hormone receptors) and/or activation of hormone-independent growth signals (e.g., tyrosine kinase cascades). In contrast, acquired resistance is mainly driven by epigenetic mechanisms. Among these, microRNAs (miRNAs) play a particularly important role, acting directly as epigenetic regulators of gene expression and indirectly via modulation of DNA methylation and histone acetylation.^6^

 To date, the involvement of miRNAs in hormonal resistance of tumors has been well documented. These include miRNAs negatively regulating estrogen receptor α (ERα) or its coactivator/corepressor proteins, as well as miRNAs controlling signaling proteins in the ERα cascade (HER2, EGFR, Akt, MAPK) and tumor suppressors.^7-16^

 In recent years, increasing attention has been paid to resistance mediated by exosomes—small extracellular vesicles secreted by cells that can be incorporated into recipient cells. In our earlier studies on estrogen-dependent MCF-7 breast cancer cells and the tamoxifen-resistant MCF-7/T subline, we demonstrated that exosomes derived from resistant cells can transfer hormonal resistance to parental cells.^17,18^

 Here, we show that stable overexpression of one exosomal miRNA—miR-181a-2—in MCF-7 cells promotes tamoxifen resistance. We observed a sustained increase in the intracellular precursor miR-181a-2 levels in cells transfected with exogenous miR-181a-2 and demonstrated the involvement of transcription factor Snail in establishing an autoregulatory loop of miR-181a-2 that maintains cell resistance. Overall, these findings reveal a novel mechanism for reprogramming resistance-associated signaling pathways via an miR-181a-2 autoregulatory loop.

## Materials and Methods

###  Cell lines and antiproliferative activity assay

 MCF-7 breast cancer cells (ATCC, Manassas, VA, USA; HTB-22) were used. Cells were cultured in DMEM (PanEco, Moscow, Russia) supplemented with glucose (4.5 g/L) and 10% fetal bovine serum (HyClone, Marlborough, MA, USA) at 37 °C in 5% CO_2_. Sensitivity to tamoxifen was evaluated using the MTT assay^19^ with modifications from the reference.^20^

###  miRNA and wtSnail transfection

 miRNA oligonucleotides were synthesized by Syntol (Moscow, Russia) and annealed in buffer (10 mM Tris-HCl pH 7.5, 50 mM NaCl, 1 mM EDTA) to obtain a 100 µM solution. Annealing was performed at 95 °C followed by slow cooling to room temperature within 1 h. Transient transfections (single and multiple) with control (scrambled) or miR-181a-2 (final concentration 50 nM) were carried out using Lipofectamine 2000 (Thermo Fisher Scientific, Waltham, MA, USA). Multiple transfections (20 rounds) were performed every three days. To suppress miR-181a-2 antisense DNA oligonucleotide with LNA-modified residues was used (the sequence ASO-181a-2-3p was the following: 5’-GG[ + T]ACAGTCAACGGTCAGT[ + G]GT-3’, [ + N] – LNA modifications) synthesized by Syntol and being transfected into the cells. For ectopic expression of wild-type Snail, we used the plasmid pcDNA3-Snail-HA kindly provided by Dr. Antonio Garcia de Herreros and the corresponding empty vector.^21^

###  Generation of cells stably expressing miR-181a-2

 The miR-181a-2 sequence was cloned into the lentiviral vector pLKO.1-TRC (Addgene #10878) following standard protocols. The miR-181a-2 gene was amplified from genomic DNA of healthy donor lymphocytes using primers with AgeI and EcoRI restriction sites. The amplified fragment was inserted into pLKO.1-TRC, and the construct was verified by Sanger sequencing.

 Lentiviral particles were produced by transfecting HEK293FT packaging cells (Thermo Fisher Scientific) with the pLKO.1-TRC-miR-181a-2 plasmid and packaging plasmids pΔR8.2 (#12263) and pVSV-G (#8454) using GenJect-39^TM^ (Molecta, Moscow, Russia). Viral supernatant was collected 24–28 h post-transfection and added to MCF-7 cells with 8 µg/mL Polybrene (Sigma-Aldrich). Infected cells were selected with 1 µg/mL puromycin for 4–5 days.

###  RNA isolation and quantitative RT-PCR

 Total RNA was extracted using TRIzol reagent (Invitrogen). cDNA synthesis was performed with Advanced cDNA Synthesis Kit (Bio-Rad) from 1 µg RNA. qRT-PCR was carried out using 5X qPCRmix-HS SYBR (Evrogen) with the following conditions: 95 °C for 3 min, followed by 40 cycles of 95 °C for 15 s, 60 °C for 15 s, and 72 °C for 30 s. Human β-actin (ACTB) served as the internal control. Relative expression levels were calculated using the ΔΔCt method.^22^

###  Immunoblotting

 Cell lysates were prepared as described previously in the reference.^23^ Proteins were separated by 10% SDS-PAGE, transferred to nitrocellulose membranes (GE Healthcare), and blocked with 5% nonfat milk (Applichem). Membranes were incubated overnight at 4 °C with primary antibodies (Cell Signaling Technology), followed by HRP-conjugated secondary antibodies (Jackson ImmunoResearch). Detection of chemiluminescence was performed using ImageQuant LAS4000 and protocol from the reference.^24^ Densitometry analysis for immunoblotting data was performed using ImageJ software (Wayne Rasband). The protocol for densitometry was provided by the University of Queensland with the recommendations from the reference.^25^

###  Reporter gene assay

 ERE-luciferase reporter activity was measured by cotransfecting cells with ERE-luciferase and β-galactosidase plasmids (control for transfection efficiency) as described in the reference.^26^ The ERE-Luc plasmid was kindly provided by George Reid and Frank Gannon.^27^ To measure Snail trans-repressor activity using E-cadherin reporter plasmid the same approach was used, the plasmid E-cadherin-Luc was kindly provided by Prof. Antonio Garcia de Herreros.^21^

###  DNA copy number quantification

 DNA copy number at the *MIR181A2HG* locus was quantified by qPCR using serial fourfold dilutions of genomic DNA to construct standard curves. *MIR181A2HG* Ct values were normalized to *ACTB*. Primer sequences are provided in Table 1.

**Table 1:** Primer sequences

**Gene**	**Forward primer (5’-3’)**	**Reverse primer (5’-3’)**	**TaqMan probe**
MIR181A2HG	GCACAGCTGCAGGGATAGTAG	GGCTGGAATTTCCTTCATTGT	FAM-GCTCTCGATCCGTGGGAGGT-BHQ1
MIR181A2 precursor	TATCAGGCCAGCCTTCAGAG	AAATCCCAAACTCACCGACA	FAM-GACTCCAAGGAACATTCAACGC-BHQ1
ACTB	ATGTGGCCGAGGACTTTGATT	AGTGGGGTGGCTTTTAGGATG	Cy5-TCATTCCAAATATGAGATGCGTTGTTACAGGA-BHQ3

###  Statistical analysis

 Experiments were performed in triplicate with three technical replicates. Data are expressed as mean ± SD. Mann-Whitney U test and unpaired t test were used to evaluate the experiments. Statistical significance was set at *P* < 0.05 (Microsoft Excel and GraphPad Prism 8).

## Results

 Previously, we demonstrated that prolonged treatment of estrogen-dependent MCF-7 breast cancer cells with exosomes derived from the estrogen-resistant MCF-7/T subline leads to a partial loss of (anti)estrogen sensitivity in MCF-7 cells.^17^ Furthermore, we showed that multiple transfections (20 sequential rounds) with one of the exosomal microRNAs, miR-181a-2, induce a similar loss of hormonal sensitivity in recipient cells, which persists for at least two months after the last transfection.^28^ Here, to further investigate the potential mechanism of miR-181a-2-induced acquired resistance, we analyzed the effects of multiple transfections on the expression of endogenous miR-181a-2 and associated signaling proteins.

###  Multiple transfections with miR-181a-2 and endogenous miR-181a-2 expression

**Figure 1:**
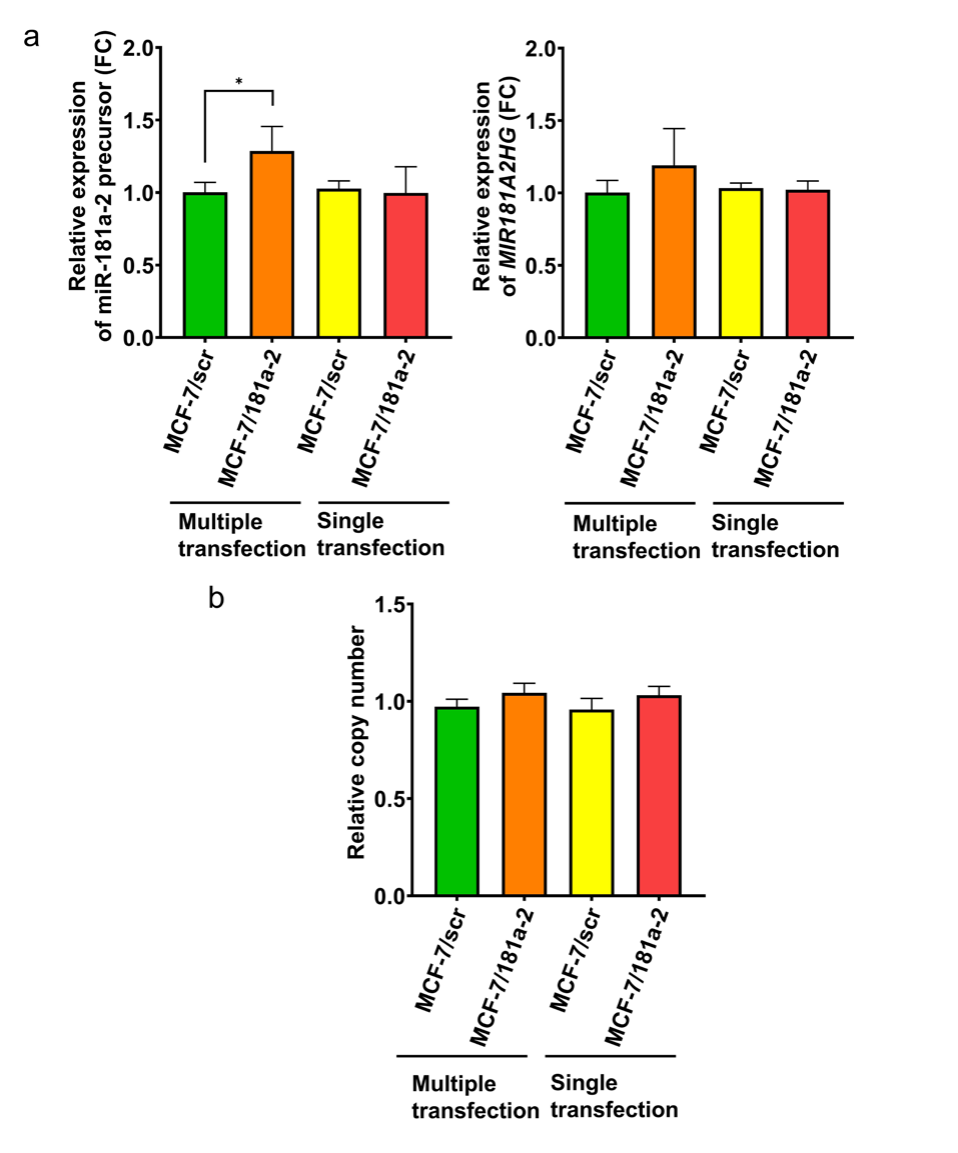
(a) Expression of cellular miR-181a-2 precursor and *MIR181A2HG *after multiple and single miR-181a-2 transfections, *p-value = 0.0286, Mann-Whitney U test, data are presented as mean ± SD (n = 3); (b) copy number of miR-181a-2-encoded DNA in MCF-7 cells after multiple and single miR-181a-2 transfections, Mann-Whitney U test, data are presented as mean ± SD (n = 3)

 Experiments were conducted on MCF-7 cells that underwent 20 rounds of miR-181a-2 transfection followed by maintenance in standard culture medium for at least two months. Specific PCR primers for the endogenous miR-181a-2 precursor were used to exclude contamination by exogenous miRNA mimetics and to evaluate only cellular miR-181a-2 expression. The data revealed a marked increase in endogenous miR-181a-2 precursor levels in multiply transfected cells. In contrast, a single transfection with miR-181a-2 did not affect the levels of endogenous miR-181a-2 (Figure 1a, left panel).

**Figure 2:**
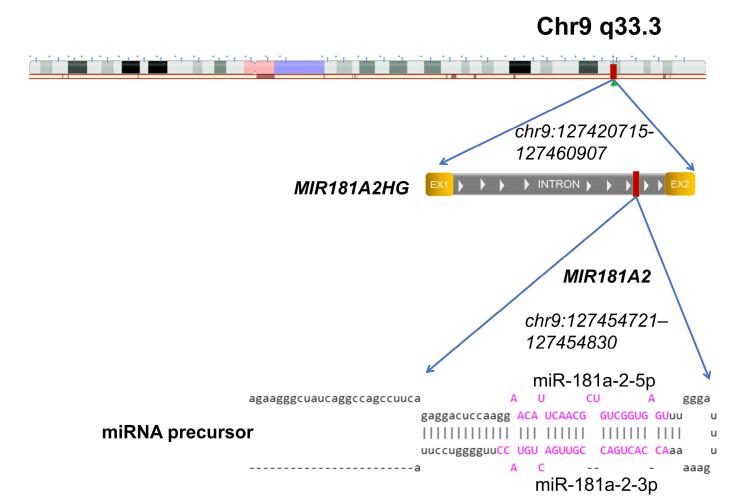
Schematic representation of the *MIR181A2HG* and *MIR181A2* genes and their genomic location. According to the UCSC Genome Browser (https://genome.ucsc.edu/, accessed 5 July 2025), *MIR181A2HG* is a long non-coding RNA that harbors the *MIR181A2* gene. Two boxes (EX1 and EX2) indicate two exons separated by an intron of the *MIR181A2HG*. The predicted secondary structure of the miR-181a-2 precursor is shown in the lower panel. The mature forms miR-181a-2-5p and miR-181a-2-3p are highlighted in purple. The *MIR181A2* gene is located within an intron of its host gene *MIR181A2HG* and is transcribed in the same direction. Genomic coordinates are provided according to the GRCh37/hg19 assembly: *MIR181A2HG* (chr9:127420715–127460907) and *MIR181A2* (chr9:127454721–127454830)

 The host gene for *MIR181A2* is *MIR181A2HG*.^29^ This gene consists of two exons and one intron and encodes a long non-coding RNA; the intron harbors the *MIR181A2* gene itself (Figure 2). It can therefore be assumed that miR-181a-2 is co-transcribed with *MIR181A2HG*. We next assessed *MIR181A2HG* transcription following single and multiple transfections with mature miR-181a-2. Similar to the precursor, *MIR181A2HG *expression increased after multiple transfections (Figure 1a, right panel).

 One potential mechanism for the elevated miR-181a-2 precursor after repeated transfections with synthetic miR-181a-2 could be amplification of the genomic region encoding *MIR181A2*. To test this, we measured DNA copy number in the *MIR181A2* locus before and after transfection. No differences were observed, excluding gene amplification as the cause of miR-181a-2 upregulation (Figure 1b).

###  MiR-181a-2 and estrogen receptor α signaling

**Figure 3:**
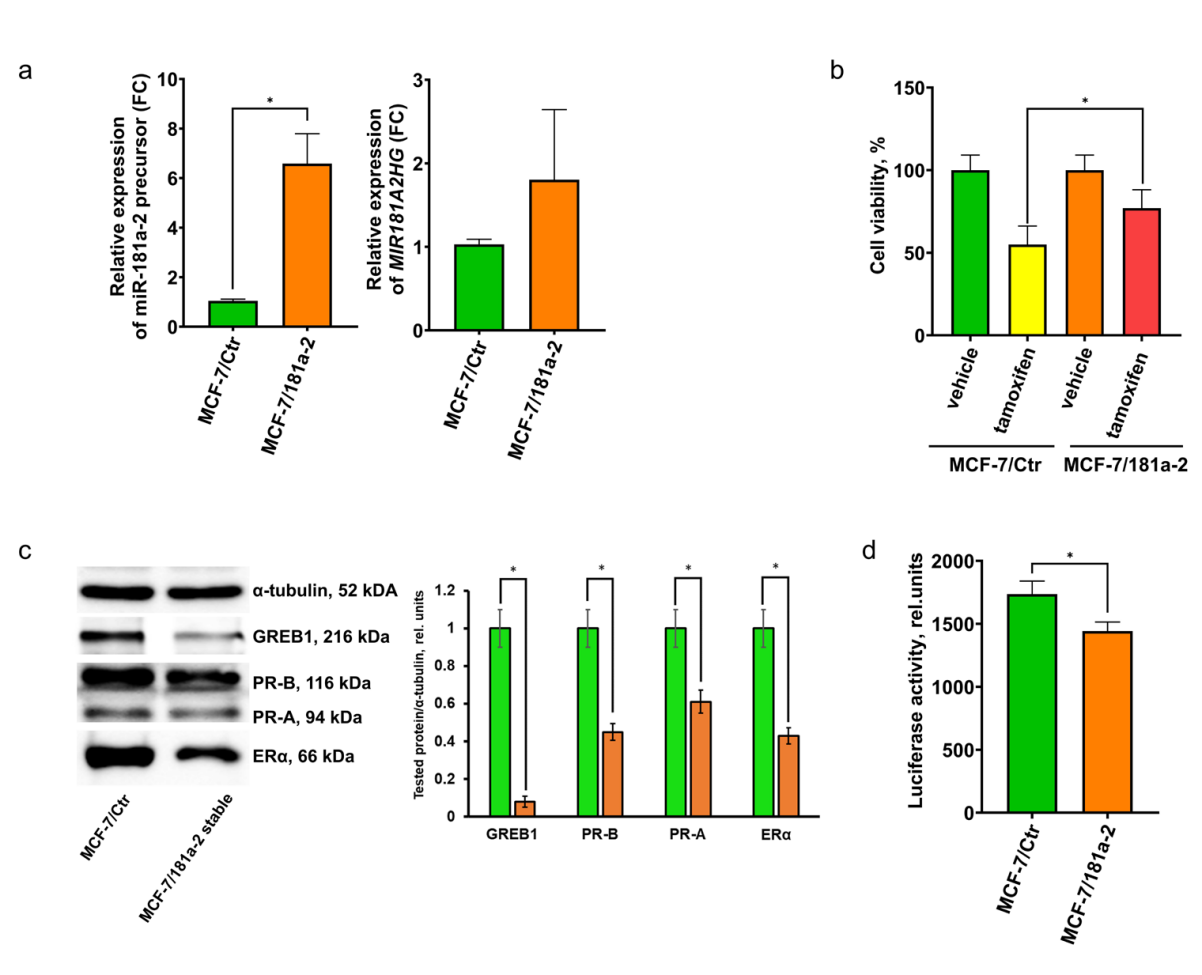
(a) Expression of cellular miR-181a-2 precursor and *MIR181A2HG* in MCF-7 cells stably infected with miR-181a-2, *P value = 0.0032, unpaired t-test, data are presented as mean ± SD (n = 3); (b) sensitivity of MCF-7/Ctr and MCF-7/miR-181a-2 cells to tamoxifen, assessed by MTT assay, *P value = 0.0317, unpaired t test, data are presented as mean ± SD (n = 3); (c) Western blot analysis of ERα-dependent proteins in MCF-7/Ctr and MCF-7/miR-181a-2 cells. The blot shows results representative of three independent experiments; t-test, *P= 0.0012 for GREB1, 0.013 for PR-B, 0.015 for PR-A, 0.031 for erα (d) reporter assay of ERα transcriptional activity in MCF-7/Ctr and MCF-7/miR-181a-2. Relative luciferase activity was calculated as the ratio of luciferase to β-galactosidase activity (arbitrary units); *P value = 0.016, unpaired t test, data are presented as mean ± SD (n = 3)

 As noted, a single miR-181a-2 transfection induces only transient estrogen resistance, whereas 20 transfections confer irreversible tamoxifen resistance lasting at least two months after the final transfection.^28^ To further investigate the effects of continuous miR-181a-2 uptake on ERα signaling, we infected MCF-7 cells with a lentiviral construct expressing miR-181a-2. Similar to multiple transfections, lentivirus-infected cells displayed stably increased levels of both the miR-181a-2 precursor and *MIR181A2HG* RNA (Figure 3a). Tamoxifen sensitivity assays revealed partial resistance in the transfected cells (Figure 3b). Moreover, miR-181a-2-overexpressing cells showed irreversible suppression of ERα signaling, including inhibition of ERα expression and transcriptional activity and downregulation of ERα-dependent proteins GREB1 and PR (Figure 3c, d). These results confirm a direct link between miR-181a-2 overexpression and ERα pathway suppression.

###  MiR-181a-2 and signaling protein expression

**Figure 4:**
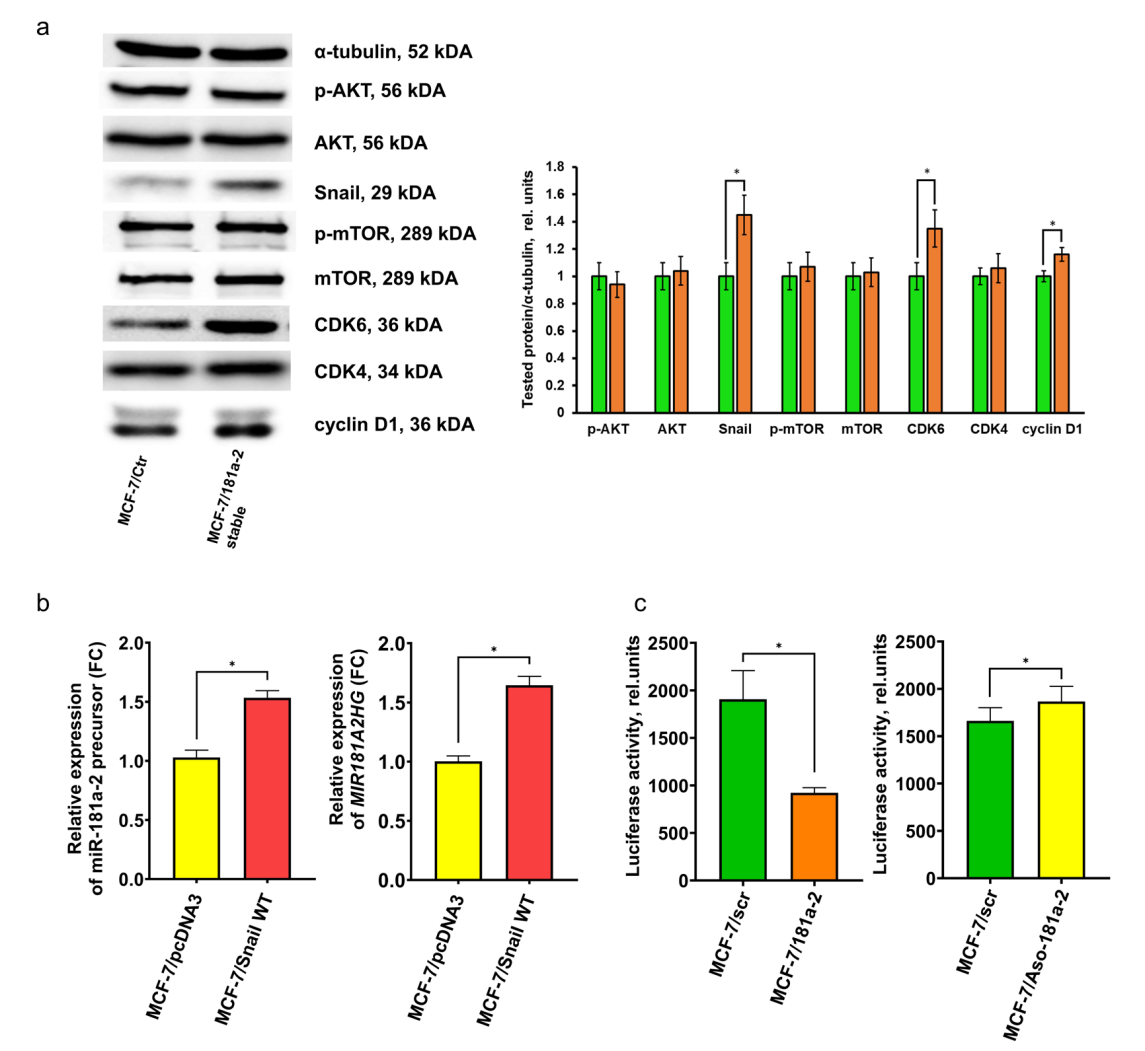
(a) Western blot analysis of growth-related proteins in MCF-7/Ctr and MCF-7/miR-181a-2 cells. The blot shows results representative of three independent experiments; t-test, *P= 0.034 for Snail, 0.035 for CDK6, 0.045 for cyclin D1 (b) expression of cellular miR-181a-2 precursor (left panel, *P value = 0.0005, unpaired t test) and *MIR181A2HG* (right panel, *P value = 0.0002, unpaired t test) in MCF-7 cells following wtSnail transfection, data are presented as mean ± SD (n = 3); (c) reporter assay of E-cadherin transcriptional activity in the cells after transfection of scrambled miR/miR-181a-2 (left panel, *P value = 0.0007, unpaired t test) and after transfection of antisense (Aso) DNA oligonucleotides scrambled/Aso-miR-181a-2 (right panel, *P value = 0.0398, unpaired t test), data are presented as mean ± SD (n = 3)

 Analysis of growth-related proteins in miR-181a-2-transfected cells revealed stable activation of CDK6, cyclin D1, and Snail, whereas major effectors of mTOR and PI3K/Akt pathways were unaffected (Figure 4a).

 To identify factors responsible for sustained endogenous miR-181a-2 overexpression, we examined the relationship between miR-181a-2 and Snail, a resistance-associated protein.^30-32^ We observed direct accumulation of endogenous miR-181a-2 in Snail-overexpressing cells, highlighting Snail’s involvement in the positive regulation and maintenance of miR-181a-2 levels (Figure 4b). Furthermore, miR-181a-2 transfection resulted in the activation of Snail trans-repressor activity (Figure 4c, left panel), whereas knockdown of miR-181a-2 using antisense (ASO) DNA oligonucleotides showed a slight tendency toward Snail suppression (Figure 4c, right panel). The modest effect observed in the latter case may be due to the relatively low number of ASO copies compared to endogenous cellular miR-181a-2 levels. Taken together, these findings suggest the possible formation of an autoregulatory loop between Snail and miR-181a-2, which may be responsible for maintenance of this signaling pathway.

 Overall, we uncovered a phenomenon of reciprocal activation: continuous uptake of exogenous miR-181a-2 mimetics induces sustained endogenous miR-181a-2 upregulation, forming an autoregulatory loop that contributes to hormonal resistance. This loop appears to be supported, at least in part, by Snail activation.

## Discussion

 Breast cancer is predominantly hormone-dependent, with estrogen receptors expressed in over 70% of cases, making them critical therapeutic targets. Hormone therapy—aimed at ERα inactivation or depletion of endogenous estrogens—is limited by the emergence of drug resistance, which can be either primary (present at diagnosis) or acquired (developing during therapy). Primary resistance is often associated with gene mutations disrupting hormonal signaling (e.g., receptor repression) or activating hormone-independent growth pathways (e.g., tyrosine kinase cascades). Acquired resistance is usually epigenomic in nature, with microRNAs playing key roles by directly suppressing specific genes or indirectly modulating DNA methylation and acetylation.

 Numerous microRNAs have been implicated in the development of hormonal resistance in tumors. These include ERα-negative regulators such as miR-342,^33^ Let-7b/Let-7i,^34^ and miR-1280.^35^ Additionally, miRNAs targeting ERα coactivators/corepressors are involved: miR-17-5p regulates SRC-3^36^; miR-10 targets the nuclear corepressor NCOR2;^16^ miR-451 regulates HER2, EGFR, and MAPK signaling^12^; and miR-101 influences Akt signaling in resistant cells.^13^ Among tumor suppressors, PTEN is particularly notable as a frequent miRNA target linked to hormonal resistance.^37-39^ Several of these miRNAs are known to be exosome-transported, supporting their role in resistance transfer.^40,41^

 Recently, increasing attention has focused on exosome-mediated resistance formation. Exosomes—microvesicles secreted by cells into the extracellular environment—can be taken up by recipient cells, transferring regulatory miRNAs that affect hormone signaling.^12,13,16,36^ These miRNAs can modulate proliferation and expression of key proteins.^42-44^ However, the duration of miRNA influence on signaling and involvement of proteins not directly targeted by specific miRNAs remain unclear. Given that miRNAs are delivered in complex mixtures containing hundreds of species, it is crucial to identify specific miRNAs and link them to defined cellular signaling changes.

 In our previous work with estrogen-dependent MCF-7 cells and tamoxifen-resistant MCF-7/T cells, we demonstrated that exosomes from resistant cells can induce hormonal resistance in parental cells. Profiling of microRNAs in “resistant” exosomes revealed overexpression of multiple ERα-targeting miRNAs, including miR-181a-2. We subsequently confirmed miR-181a-2 as a key driver of tamoxifen resistance.^28^ These findings are consistent with other studies linking miR-181 to drug resistance in various models.^45-47^ Nonetheless, the mechanisms underlying long-term maintenance of resistance in newly generated resistant cells remain unclear.

 Here, for the first time, we describe hyperexpression of endogenous miR-181a-2 precursor in MCF-7 cells following multiple (20 rounds) transfections with exogenous miR-181a-2 mimetics. Elevated precursor levels persisted for at least two months post-transfection. Multiply transfected cells exhibited partial hormonal insensitivity, suppression of estrogen signaling, DNMT3A inhibition, and increased expression of growth-related proteins, consistent with known properties of miR-181.^14,47-50^

 Among these proteins, we identified Snail—an EMT regulator and indirect miR-181 target. Our results are consistent with the data of other researchers who have demonstrated the increase in Snail level in the miR-181-overexpressed cells.^51-53^ Partially, the recent observations have shown the ability of miR-181a, along with TMBIM6 protein, to increase Snail level through the activation of ERK pathway in breast cancer cells.^51^

 We and others have previously shown that Snail contributes to resistance to hormonal drugs.^30-32^ Consistent with this, we observed an association between Snail overexpression and elevated miR-181a-2 precursor levels, implicating Snail as a key mediator of miR-181a-2-induced resistance. Finally, we have demonstrated the formation of autoregulatory loop between Snail and mir-181a-2 that may be responsible for cell resistance phenotype.

## Conclusion

 We propose a mechanism of cancer cell resistance based on an autoregulatory loop sustaining high levels of resistance-associated factors, such as miR-181a-2. Snail plays a central role in this loop: it both activates miR-181a-2 and is itself a target of miR-181a-2. The precise mediators transmitting signals from Snail to miR-181a-2, the mechanisms by which this loop is preserved during cell division, and its role in biochemical imprinting and broader phenotypic adaptation of cancer cells remain open questions for future investigation.

## Competing Interests

 All authors declared that there are no conflicts of interest.

## Consent for publication

 Not applicable.

## Data Availability Statement

 The datasets of the current study are available from the corresponding author upon request.

## Ethical Approval

 Not applicable.
